# The effect of past use of oral contraceptive on bone mineral density, bone biochemical markers and muscle strength in healthy pre and post menopausal women

**DOI:** 10.1186/1472-6874-9-31

**Published:** 2009-11-03

**Authors:** Fadoua Allali, Laila El Mansouri, Fatima zohra Abourazzak, Linda Ichchou, Hamza Khazzani, Loubna Bennani, Redouane Abouqal, Najia Hajjaj-Hassouni

**Affiliations:** 1 Department of Rheumatology, El Ayachi University-Hospital, Sale, Morocco; 2 LIRPOS: Laboratory of information and research on bone diseases, Faculty of Medicine and Pharmacy, Rabat, Morocco; 3 Laboratory of Biostatistical, Clinical Research and Epidemiology (LBRCE), Faculty of Medicine and Pharmacy, Rabat, Morocco

## Abstract

**Background:**

during adulthood, most studies have reported that oral contraceptive (OC) pills had neutral, or possibly beneficial effect on bone health. We proposed this study of pre and post menopausal women assessing BMD, bone biochemical markers and physical performance among OC past users and comparable women who have never use Ocs.

**Methods:**

A cross-sectional study comparing the bone density, bone biochemical markers (osteocalcin, CTX) and three measures to assess physical performance: timed get-up-and-go test "TGUG", five-times-sit-to-stand test "5 TSTS" and 8-feet speed walk "8 FSW" of users and never users OC. We were recruited 210 women who used OC for at least 2 years with that of 200 nonusers was carried out in pre and postmenopausal women (24-86 years).

**Results:**

when analysing the whole population, BMD and biochemical markers values were similar for Ocs past users and control subjects. However when analysing the subgroup of premenopausal women, there was a statistically significant difference between users and never-users in osteocalcin (15,5 ± 7 ng/ml vs 21,6 ± 9 ng/ml; p = 0,003) and CTX (0,30 ± 0,1 ng/ml vs 0,41 ± 0,2 ng/ml; p = 0,025). This difference persisted after adjustment for age, BMI, age at menarche and number of pregnancies. In contrast, in post menopausal women, there was no difference in bone biochemical markers between OC users and the control. On the other hand OC past users had a significant greater performance than did the never users group. And when analysing the physical performance tests by quartile OC duration we found a significant negative association between the three tests and the use of OC more than 10 years.

**Conclusion:**

the funding show no evidence of a significant difference in BMD between Ocs users and never user control groups, a decrease in bone turn over in OC pre menopausal users and a greater physical performances in patients who used OC up than 10 years.

## Background

Osteoporosis in women following menopause is a major health problem that leads to a high incidence of spine and hip fractures. Many risk factors contribute to its development and it is now well recognized that a chronically hypoestrogenic state increases bone turnover that, in turn, causes a critical decrease in bone mineral density (BMD), an important determinant of fracture risk. Bone loss following menopause can be prevented or reduced by replacement hormonal therapy.

Considerable controversy exists, however, as to whether Oral contraceptives (Ocs) possess positive influences on bone. Ocs have been reported to be a protective agent against low BMD in several studies [1-4], but there are also conflicting results [5,6]. In a review article Kuohung [7] could not find any consensus on whether or not Ocs use had a protective effect on BMD and bone metabolism. Regarding the premenopausal women, there are although a relatively few cross-sectional studies of OC use and BMD have focused on premenopausal women. The Canadian multicenter osteoporosis study found lower BMD values in spine and trochanter in premenopausal women who had used Ocs compared with those who have never used Ocs [8]. A large study in a selected population of premenopausal Finnish women showed a small positive correlation between OC use and age adjusted DXA measurements [9]. These conflicting findings may be due in large part the oestrogen/progestin doses in the OC preparations, the duration of the studies and anatomic sites evaluated.

On the other hand, previous research examining the influence of oral contraceptive use on muscle function has been limited as pill users have been used as a control rather than an experimental group. Sarwar and al demonstrated changes in strength, relaxation and fatigability in human muscle during the menstrual cycle while there were no changes in any parameter in the women taking the OC [10]. Elliott et al suggested that oral contraceptive use does not significantly affect muscle strength [11].

We proposed a cross sectional study of pre and post menopausal women assessing BMD, bone biochemical markers and physical performance among OC past users and comparable women who have never use Ocs.

## Methods

### Design and subjects

We conducted a cross-sectional study of 210 women who had used combined OC for at least 2 years and compared them to 200 who had never used OC. Women selected for the study were recruited from the city of Rabat, through advertisements in local hospitals. Informed consent was obtained from all subjects and the study was approved by ethics committee of our university hospital. We excluded from the study all patients with a history of: (1) taking drugs known to influence bone metabolism in the past 2 years, such as vitamin D, calcium, corticosteroids, bisphosphonates and hormone replacement therapy; (2) musclo-skeletal, thyroid, parathyroid, adrenal, hepatic, or renal disease; (3) malignancy; and (4) hysterectomy.

### Data collection and measurements

Each patient completed a questionnaire on socio demographic parameters and osteoporosis risk factors including section on obstetric and menstrual history. Reproductive history included age at menarche, number of pregnancies, duration of lactation and age at menopause. Oral contraceptives use was documented by the age at which a women initiated use, and the name and type of each preparation with the period of its use.

### Bone mineral density measurements (BMD)

Lumbar spine, trochanter, femoral neck and total hip BMD were measured by dual-energy X-ray absorptiometry with a Lunar prodigy densitometer. Daily quality control was carried out by measurement of a Lunar phantom. At the time of the study, phantom measurements showed stable results. The phantom precision expressed as the CV(%) was 0.08. Both T and Z scores were obtained. In the T-score calculations, the manufacturer's ranges for European reference population were used because of the absence of a Moroccan data base.

### Biochemical measurements

Morning fasting blood and random urine samples were collected from every subject for the measurement of the following parameters: serum calcium, phosphorus, albumin, creatinine, 25(OH) D, osteocalcin, and C-terminal cross-linking telopeptide of type I collagen (CTX). Serum calcium, phosphorus, creatinine and albumin were measured by automated standard laboratory methods. Serum 25(OH) D was measured by chemiluminescence (Liaison, Diasorin). The intra- and interassay coefficients of variation (CVs) were 5% and 11%, respectively and the normal range was 20-60 ng/ml. Osteocalcin and CTX were both measured by immunochemoluminometric assay (Elecsys, Roche diagnostics, Mannheim, Germany). Intra- and interassay variances were 5% and 7% and the normal range were 15--46 ng/ml for osteocalcin, and 0.3 ng/ml-0.6 ng/ml for CTX.

### Physical Performance Measures

Three measures to assess physical performance were used: timed get-up-and-go test, five-times-sit-to-stand test and 2.4 meters speed walk. Time was measured in seconds with a stopwatch and rounded to the nearest hundredth of a second.

#### Timed get-up-and-go test (TGUG)

Time taken to complete the test "Get-up-and-Go": the subject rises from a chair, walks 3 meters, turns around, returns to the chair, and sits down. The subject was instructed to: "Sit with your back against the chair and your arms on the arm rests. On the word 'go', stand upright, then walk at your normal pace to the line on the floor, turn around, return to the chair, and sit down." The stopwatch was started on the word 'go' and stopped when the subject returned to the starting position.

#### Five-times-sit-to-stand test (5 TSTS)

Participants were asked to stand up and sit down five times as quickly as possible without the use of hands and were timed from their initial sitting position to the final standing position at the end of the fifth stand.

#### 8-feet (2.4 meters) speed walk (8 FSW)

The women were instructed to walk as fast as they could in their ordinary shoes for 2.4 meters. Participants used the footwear they normally used. The distance was marked on the floor with red tape and the participant stood just behind the starting line before the test. A digital stopwatch was started when the participant started to walk and stopped when the first foot crossed the finishing line.

### Statistical analysis

The primary analysis of this study compared women who used Ocs up than 2 years with those who had never used Ocs. We used the Student's *T test *for matched samples and the *Khi-2 test *for the analysis of qualitative variables. Descriptive statistics are presented as means and standard deviations (SDs) for continuous variables. The factors that remained significant or had a strong association with the biochemical markers, BMD or physical tests were tested by multiple linear regression analysis to eliminate potentially confounding factors (age, BMI, number of pregnancies, age at menarche,25 OH Vit D, total calcium intake). All analyses were performed using SPSS, version 10.0 for Windows. Results with *p *values < 0.05 were considered statistically significant.

## Results

In Table 1 we summarize the distribution of demographic and other characteristics of OC past users and the control group. In the total study population (pre and post menopausal), the mean duration of OC use was 7,7 ± 6 years with a range of 2 to 30 years. OC past users were likely than women who had never use contraception to be younger (54,3 ± 7,7 years vs 57,2 ± 11,2 years p = 0,003). The groups were similar in terms of BMI, daily calcium intake and age at menarche. Both before and after adjustment for covariates, pre and post menopausal women using Ocs and comparisons women did not differ significantly in mean BMD at the lumbar spine, Trochanter, femoral neck and total femur (table 1). Duration of OC exposure (< 2-4 years; 4-6 years; > 6 years) vs no hormonal contraception did not alter this finding (result not shown).

**Table 1:** Demographic and anthropometric characteristics of Ocs users and nonusers

	**All patients** **n = 410**		**Pre-menopausal women n = 92**		**Post menopausal women n = 318**	
**Variables**	**OC users** **N = 210**	**Nonusers** **n = 200**	**OC users** **n = 43**	**Nonusers** **n = 49**	**OC users** **n = 167**	**Nonusers** **n = 151**

Age (years)	54.3 ± 7.7	57.2 ± 11.2*	45 ± 5	43.9 ± 6.3	56.7 ± 6.3	61.5 ± 8.8
BMI (kg/m2)	28 ± 4.5	28.5 ± 4.8	28.8 ± 3.9	27.2 ± 4.3	27.8 ± 4.6	28.8 ± 4.9
Age at menarche (years)	12.7 ± 1.8	12.6 ± 1.6	13.1 ± 1.6	12.4 ± 1.4	12.5 ± 1.8	12.6 ± 1.7
Number of pregnancies	4.4 ± 2.2	4.2 ± 3.5	3.6 ± 1.8	1.6 ± 1.4	4.6 ± 2.2	5 ± 3.5
Breast feeding duration (months)	29.9 ± 42.7	30 ± 42.9	17.5 ± 21.9	10.5 ± 12	33.1 ± 46	35.9 ± 47
Age of menopause (years)	46.5 ± 9.4	47 ± 6.8	-	-	47.9 ± 4.9	47.3 ± 5.7
Daily calcium intake (mg/j)	695 ± 229	688 ± 226	730 ± 258	713.1 ± 204	686.9 ± 221	680.8 ± 233
Lumbar spine(g/cm2)	1.01 ± 0.17	0.99 ± 0.19	1.14 ± 0.13	1.11 ± 0.17	0.984 ± 0.16	0.964 ± 0.18
Femoral neck (g/cm2)	0.88 ± 0.13	0.88 ± 0.15	1 ± 0.13	0.9 ± 0.15	0.859 ± 0.12	0.852 ± 0.14
Word's triangle (g/cm2)	0.72 ± 0.16	0.71 ± 0.17	0.86 ± 0.13	0.81 ± 0.15	0.69 ± 0.15	0.68 ± 0.16
Trochanter (g/cm2)	0.72 ± 0.12	0.71 ± 0.14	0.82 ± 0.1	0.77 ± 0.13	0.699 ± 0.11	0.693 ± 0.13
Total femur (g/cm2)	0.93 ± 0.14	0.91 ± 0.15	1.06 ± 0.1	1.01 ± 0.1	0.903 ± 0.13	0.884 ± 0.14
Calcium (mg/dl)	96.4 ± 5.2	97 ± 4.8	95.4 ± 6.1	95.9 ± 3.9	96.9 ± 4.7	97.6 ± 5.27
Phosphatus (mg/dl)	36.1 ± 4.9	35.5 ± 5	34.56 ± 5.13	34.08 ± 4.16	36.9 ± 4.7	36.4 ± 5.3
25 OH Vitamin D	18.8 ± 8.1	17.4 ± 7.9	20.2 ± 7.4	17.08 ± 7.8	17.7 ± 8.17	18.16 ± 8.46
Osteocalcin (ng/ml)	21.4 ± 10	23.9 ± 14	15.5 ± 7.5	21.6 ± 9*	23.9 ± 10.4	25.4 ± 17.4
CTX (ng/ml)	0.42 ± 0.2	0.46 ± 0.21	0.30 ± 0.11	0.41 ± 0.2*	0.47 ± 0.22	0.50 ± 0.29

*Significantly different from Oral contraception users. p < 0,005

When analysing the whole population, biochemical markers values were similar for Ocs past users and control subjects: osteocalcin (21,4 ± 10 ng/ml vs 23,9 ± 14 ng/ml; p = 0,1) and CTX: (0,42 ± 0,2 ng/ml vs 0,46 ± 0,2 ng/ml; p = 0,1) respectively (table 1). However, when analysing the subgroup of premenopausal women, there was a statistically significant difference between OC past users and never-users in osteocalcin (15,5 ± 7 ng/ml vs 21,6 ± 9 ng/ml; p = 0,003) and in CTX (0,30 ± 0,1 ng/ml vs 0,41 ± 0,2 ng/ml; p = 0,025). This difference persisted after adjustment for the important variables relating to contraception and biochemical markers: age, BMI, age at menarche and number of pregnancies (table 2).

**Table 2:** Relationship between oral contraception use and biochemical bone markers in linear regression analysis

	**CTX**	**Osteocalcin**			
	**β**	**P**	**β**	**SD**	**p**

Age	0,15 ± 0,23	0,5	0,01	0,01	0,09
BMI	-0,18 ± 0,31	0,5	0,002	0,01	0,7
Age at menarche	0,91 ± 0,80	0,2	0,02	0,02	0,3
Number of pregnancies	0,33 ± 0,68	0,6	0,002	0,02	0,8
Oral Contraception	-7,71 ± 2,80	0,01	-0,12	0,06	0,03

### Association between oral contraception use and physical performances

**Figure 1:**
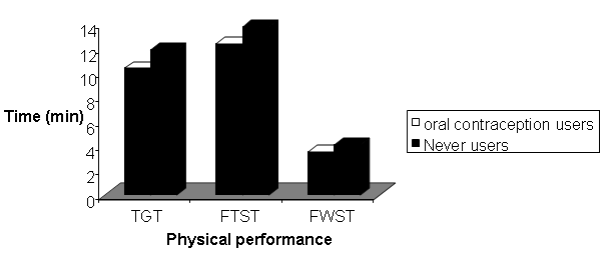
**Comparison of mean duration of the three physical performance tests**.

A comparison of mean duration of the three physical performance tests according to the use of OC is shown in figure 1. OC past users had a significant greater performance than did the never users group. This difference persisted after adjusting for age.

The crude and adjusted mean differences for the timed get-up-and-go test, the 8-foot walk test and the sit-to-stand test by quartile OC duration are shown in Table 3. The never user group was use a reference. There was a significant negative association between the Timed get-up-and-go test and the use of OC more than 10 years in the crude analysis (P: 0,002) and after control for demographic variables including age, menopausal status and 25 OH vitamin D level (P: 0,027). Similar results were observed with the sit-to-stand test. In the adjusted analysis, subjects who used OC more than 10 years were 1.84 s quicker in the Timed get-up-and-go test and 1.9 s quicker in the sit-to-stand test (both: P < 0,005) than were never user subjects. For the 8-foot walk test, there was also a significant negative relation with the use of OC up than 10 years, but after adjustment for age, menopause and 25 OH vitamin D level the effect of OC on the 8FWt was substantially diminished and no longer achieved statistical significance.

**Table 3:** Relationship between physical test and oral contraception duration use.

	**Univariabl analysis**	Multivariabl analysis*
	**β (CI 95)**	**β (CI 95)**
Timed get-up-and-go test		
Never user	0 (reference)	0 (reference)
OC use < 3 y	-1.39 (-3.31, 0.62)	-0.89 (-2.7,0.9)
OC use for 3-6 y	0.04 (-2.13, 2.22)	-0.45 (-2.5, 1.6)
OC 6-10 years	0.42 (-1.98, 2.83)	-0.32 (-2.5, 1.8)
OC > 10 years	-2.52 (-4.15,-0.93)	-1.84 (-3.2,-0.47)
Five-times-sit-to-stand test		
Never user	0 (reference)	0 (reference)
OC < 3 years	-1.39 (-3.30, 0.50)	-1.62 (-4,0.7)
OC 3-6 years	0.41 (-1.67, 2.49)	0.79 (-1.8, 3.4)
OC 6-10 years	-0.54 (-2.84, 1.87)	0.34 (-2.4, 3.1)
OC > 10 years	-1.56 (-3.10, -0.04)	-1.9 (-3.6, -0.17)
8-feet (2.4 meters) speed walk		
Never user	0 (reference)	0 (reference)
OC < 3 years	-0.42 (-1.21, 0.37)	0.07 (-0.7,0.9)
OC 3-6 years	-0.21 (-1.07, 0.65)	-0.59 (-1.5, 0.3)
OC 6-10 years	-0.014 (-0.97, 0.94)	-0.3 (-1.3, 0.7)
OC > 10 years	-0.85 (-1.50, -0.22)	-0.22 (-1.3,-0.7)

* adjustment on age, menopausal status and 25 OH Vit D.

## Discussion

The findings of this cross-sectional study of pre and post menopausal women suggest no evidence of a significant difference in BMD between the contraceptive past users and never user control groups. This result is in agreement with some studies that have found no effects of OC on BMD [5,12]. Available data on the effect of OC on BMD are contradictory: Some previous studies found a positive [1-4] association between BMD and the use of OC, whereas others observed a lack of association [7,13]. The apparently divergent findings from these previous studies could be attributed to the wide range of ages studied, the duration of the studies and the dosage of Ocs. Indeed, Horsman and al reported that postmenopausal women taking doses of oestrogen (EE) between 15 and 25 mg daily experienced no bone loss, whereas those taking doses of > 25 mg daily demonstrated net gain of bone [14]. Thus, improved bone mineralization among low-dose OC users is biologically plausible. Furthermore, conflicting finding may be due in large part to the longer duration of OC use. Berenson and al reported that long-term use of oral contraceptives increased bone mass [15]. This fact was supported by some studies showing that the high-dose OC use for more than 10 years had the greatest protection against low BMD [16,17]. Nevertheless, Petitti reported that oral contraceptives had only short-term effect in increasing BMD and this effect was reversible [12]. But the mechanism involved in reversing a hormonally initiated loss of BMD is not clear. In our study, the nature and the exact dosage of Ocs used was not known. But in the 1960s, when the women of our study needed contraception, the oestrogen content of Ocs has always been 50 ug ethinyl estradiol or less. Furthermore, longer duration of OC use didn't show any influence on BMD.

Cross-sectional and prospective previous investigation of biochemical markers of bone turnover in adult OC users has produced findings consistent with those in our study of decreased bone turnover. Garnero and al examined several biochemical markers, including BSAP and DPD in a cross-sectional comparison of users and nonusers of Ocs [18]. When compared to the nonusers, the markers of bone formation in the OC users decreased 15--24% and bone resorption decreased 17--28%. Utilizing a prospective design, Karlsson and al reported a 50% decrease in osteocalcin in OC users after 3 months [19]. In our study, bone turn over markers (osteocalcin and CTX) were decreased in premenopausal women who used OC compared with none users while there was no change in post menopausal women.

The effects of OC on muscle mass and performance have been less well investigated. Exogenous, synthetic reproductive hormones (in particular HRT) have been shown to increase strength [20,21]. Therefore strength might have been expected to increase as a result of oral contraceptive administration. However, there are conflicting data regarding the effect of OC on physical performances, which may be due partly to differences in the treatment combinations of oestrogen and progestin. OC use has been shown to decrease, maintain, or have no effect on a variety of strength measures [22-25]. In the present study, patients who used oral contraceptive up than 10 years had better physical performances than non users. Despite adjustment for major confounding factors, the result did not fluctuate. However, this effect of OC on physical performance should be viewed with caution, as we are not aware of any literature at present that finds the same results.

Our study has many limitations. First, it was a cross sectional study which the information was limited to past use and wholly based on the subjects' recall instead of medical records and no information on type of Ocs was available. Secondly, although the overall sample size was adequate, the number of subjects in premenopausal women was small, that may could reduced our power to detect small differences in BMD.

However, this study had a number of strengths. First, the study consisted of a large sample size. Second, we evaluated three criteria in the same study; bone mineral density, biochemical markers of bone turnover and physical performance with the use of a variety of validated test measures. Finally, all variables were analyzed in two distinct groups: pre and post menopausal women.

## Conclusion

In conclusion, the finding of this cross-sectional study of pre and post menopausal women show no evidence of a significant difference in BMD between Ocs users and never user control groups, a decrease in bone turn over in OC pre menopausal users and a greater physical performances in patients who used OC up than 10 years. Future research should investigate the effect of oral contraceptive use on other strength and performance based parameters (such as muscle fatigability/endurance trials) and various health measures so that specific recommendations can be made to the pill user.

## Cometing interests

The authors declare that they have no competing interests.

## Authors' contributions

-We declare that we participated at the study as following:

FA, NH-H and RA conceived the the original idea for the study and supervised its design, execution, and analysis and participated in the drafting and critical review of the manuscript. FA and RA did data management and statistical analyses. LE participated in study design, wrote the paper with input from all investigators. All other authors enrolled patients, participated in data acquisition and critical revision of the manuscript

All authors read and approved the final manuscript.

## Pre-publication history

The pre-publication history for this paper can be accessed here:


